# Blood pressure control in diabetic kidney disease: a post-hoc analysis of the FANTASTIC trial

**DOI:** 10.1186/s40885-024-00280-x

**Published:** 2024-08-01

**Authors:** Cheol Ho Park, Soon Jun Hong, Sung Gyun Kim, Seok Joon Shin, Dong Ki Kim, Jung Pyo Lee, Sang Youb Han, Sangho Lee, Jong Chul Won, Young Sun Kang, Jongha Park, Byoung-Geun Han, Ki-Ryang Na, Kyu Yeon Hur, Yong-Jin Kim, Sungha Park, Tae-Hyun Yoo

**Affiliations:** 1 Department of Internal Medicine, Institute of Kidney Disease Research, Yonsei University College of Medicine, Seoul, Republic of Korea; 2 Department of Cardiology, Korea University Anam Hospital, Seoul, Republic of Korea; 3 Division of Nephrology, Hallym University Sacred Heart Hospital, Anyang, Republic of Korea; 4 Division of Nephrology, Incheon St. Mary’s Hospital, The Catholic University of Korea, Incheon, Republic of Korea; 5 Department of Internal Medicine, Seoul National University College of Medicine, Seoul, Republic of Korea; 6 Department of Internal Medicine, Seoul National University Boramae Medical Center, Seoul, Republic of Korea; 7 Division of Nephrology, Inje University Ilsan-Paik Hospital, Goyang, Republic of Korea; 8 Department of Nephrology, Kyung Hee University Hospital at Gangdong, Seoul, Republic of Korea; 9 Division of Endocrinology and Metabolism, Inje University Sanggye Paik Hospital, Inje University School of Medicine, Seoul, Republic of Korea; 10 Division of Nephrology, Korea University Ansan Hospital, Ansan, Republic of Korea; 11 Division of Nephrology, Ulsan University Hospital, Ulsan, Republic of Korea; 12 Division of Nephrology, Yonsei University Wonju College of Medicine, Wonju, Republic of Korea; 13 Division of Nephrology, Chungnam National University Hospital, Daejeon, Republic of Korea; 14 Division of Endocrinology and Metabolism, Samsung Medical Center, Sungkyunkwan University School of Medicine, Seoul, Republic of Korea; 15 Division of Cardiology, Seoul National University Hospital, Seoul, Republic of Korea; 16 Division of Cardiology, Severance Cardiovascular Hospital, Cardiovascular Research Institute, Yonsei University College of Medicine, Seoul, Republic of Korea

**Keywords:** Blood pressure, Cardiovascular outcome, Diabetic kidney disease, Kidney outcome

## Abstract

**Background:**

The target blood pressure (BP) value is unclear for diabetic kidney disease (DKD). Therefore, we aimed to evaluate the effect of strict BP control or ‘on treatment’ BP on clinical outcomes in patients with DKD.

**Methods:**

A *post-hoc* analysis of the prespecified secondary outcomes of the FimAsartaN proTeinuriA SusTaIned reduCtion in comparison with losartan in diabetic chronic kidney disease (FANTASTIC) trial, a randomized multicenter double-blind phase III trial. Eligible patients were aged ≥ 19 years with DKD. We assigned 341 participants with DKD to BP control strategy (standard-systolic BP [SBP] < 140 mmHg *versus* strict-SBP < 130 mmHg). The outcome was the occurrence of cardiovascular events and renal events. Separate analyses were performed to compared the risk of outcome according to achieved average BP levels.

**Results:**

A total of 341 participants were included in the analysis. Over a median follow-up of 2.8 years, cardiovascular/renal events were observed in 25 (7.3%) participants. Mean (SD) SBPs in the standard and strict BP control group were 140.2 (11.6) and 140.2 (11.9) mmHg, respectively. The strict BP control group did not show significantly reduced risk of cardiovascular/renal events (HR 1.32; 95% CI 0.60–2.92]). In the post-hoc analyses using achieved BP, achieved average SBP of 130–139 mmHg resulted in reduced risk of cardiovascular/renal events (HR 0.15; 95% CI 0.03–0.67) compared to achieved average SBP ≥ 140 mmHg, whereas further reduction in achieved average SBP < 130 mmHg did not impart additional benefits.

**Conclusion:**

In patients with DKD, targeting a SBP of less than 130 mmHg, as compared with less than 140 mmHg, did not reduce the rate of a composite of cardiovascular and renal events. Achieved SBP of 130–139 mmHg was associated with a decreased risk for the primary outcome in patients with DKD.

**Trial registration:**

ClinicalTirals.gov Identifier: NCT02620306, registered December 3, 2015.

(https://clinicaltrials.gov/study/NCT02620306).

**Supplementary Information:**

The online version contains supplementary material available at 10.1186/s40885-024-00280-x.

## Introduction

Recently, the Systolic Blood Pressure Intervention Trial (SPRINT) demonstrated that targeting a systolic blood pressure (SBP) < 120 mmHg, as compared to < 140 mmHg, was superior in reducing adverse cardiovascular events and all-cause death [1]. In a subgroup analysis of patients with non-diabetic chronic kidney disease (CKD) enrolled in the SPRINT study, intensive BP lowering reduced the rates of major cardiovascular events and death from any cause, without deleterious effect on kidney function [2]. These findings were adopted in the 2021 Kidney Disease: Improving Global Outcomes (KDIGO) practice guidelines for blood pressure (BP) managements that recommend a target SBP < 120 mmHg in CKD patients [3].

However, in patients with diabetic kidney disease (DKD), whether intensive BP control results in favorable outcomes including adverse kidney events, cardiovascular events, or all-cause death remains unclear [4–7]. This uncertainty may be attributed to the exclusion of diabetic patients from the SPRINT study and the paucity of randomized controlled trials that exclusively investigate the effect of intensive BP control in DKD patients [1, 3–5]. The Action to Control Cardiovascular Risk in Diabetes (ACCORD) study that evaluated the effect of a lower SBP target of 120 mmHg on cardiovascular events compared with that associated with a conventional SBP target of 140 mmHg in patients with type 2 diabetes, failed to prove any benefits of intensive BP control [8, 9]. Furthermore, an SBP goal < 120 mmHg even increased the risk of adverse kidney events [8, 9]. However, ACCORD study participants’ mean estimated glomerular filtration rate (eGFR) and median urine albumin-to-creatinine ratio (UACR) were 91.6 mL/min/1.73 m^2^ and 14 mg/gCr, respectively; these participants were unlikely to be representative of DKD patients [8].

The FimAsartaN proTeinuriA SusTaIned reduCtion in comparison with losartan in diabetic chronic kidney disease (FANTASTIC) clinical trial evaluated the efficacy of angiotensin receptor blockers (ARBs: fimasartan *versus* losartan) and lowering BP (SBP target: < 130 mmHg *versus* < 140 mmHg) in reducing albuminuria in patients with DKD [10]. This trial offers an opportunity to elucidate the uncertainty regarding BP target values in DKD patients. Our study aimed to evaluate the effect of strict BP control or ‘on treatment’ BP on clinical outcomes in patients with DKD.

## Methods

### Study design and participants

This study reports the prespecified secondary outcomes from as well as a *post-hoc* analysis of the FANTASTIC trial (ClinicalTirals.gov Identifier: NCT02620306, registered December 3, 2015, https://clinicaltrials.gov/study/NCT02620306), a randomized multicenter double-blind phase III trial conducted at 34 clinical sites in the Republic of Korea between 2016 and 2022. The FANTASTIC trial enrolled 351 adults. Patients with hypertensive diabetic CKD with overt albuminuria were eligible for screening. The inclusion criteria were: 1) adults aged 19 years or older with type 2 diabetes mellitus diagnosed at least 3 months before screening without any changes in the dose of medications for at least 3 months; 2) average BP for treatment-naive patients: 140 mmHg ≤ SBP < 180 mmHg and diastolic BP (DBP) < 110 mmHg, and for patients who received an angiotensin converting enzyme inhibitor (ACEi) or ARB: 130 mmHg ≤ SBP < 180 mmHg and DBP < 110 mmHg; 3) estimated glomerular filtration rate (eGFR): ≥ 30 mL/min/1.73 m^2^ within the past 6 months; and 4) UACR within the past 12 months that met one or more of the following conditions: UACR > 300 mg/g or at least two results of 30 ≤ UACR ≤ 300 mg/g with an interval between the two tests of at least 12 weeks. The exclusion criteria were: 1) severe hypertension with average SBP ≥ 180 mmHg or DBP ≥ 110 mmHg; 2) symptomatic orthostatic hypotension; 3) insulin-dependent type 1 diabetes mellitus or uncontrolled diabetes mellitus; 4) patients undergoing dialysis; or 5) patients with clinically significant decompensated cardiac and hepatic diseases. Details about the FANTASTIC trial can be found elsewhere [10, 11].

**Fig. 1:**
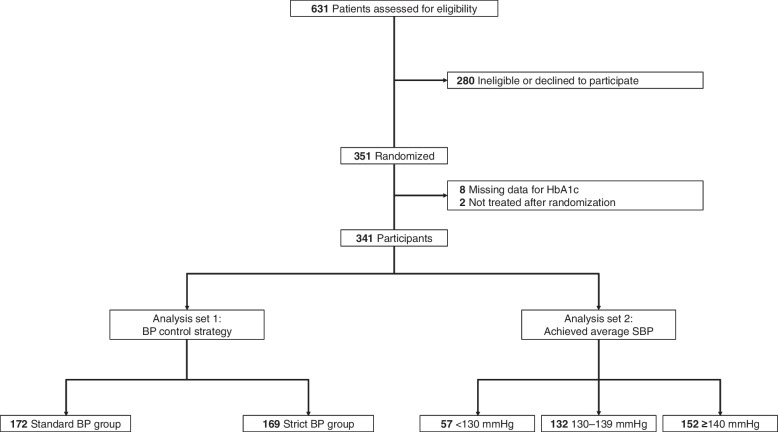
The flow of the study. Abbreviations: BP, blood pressure; eGFR, estimated glomerular filtration rate; SBP, systolic blood pressure

We excluded eight participants with missing information regarding HbA1c and two who were not treated after randomization, resulting in a final analysis of 341 participants (Fig. 1). The participants were followed-up until May 2022.

The study was conducted in accordance with the principles of the Declaration of Helsinki. All participants provided written informed consent based on the documents approved by each participating centers’ Institutional Review Board (Severance Hospital: 4–2015-0848).

### Randomization and study procedures

After screening, eligible subjects were equally randomized into one of four groups (fimasartan group A, fimasartan group B, losartan group A, and losartan group B). Group A had standard BP control (SBP < 140 mmHg), while group B had strict BP control (SBP < 130 mmHg). The stratified block randomization employing each participating site as a stratification factor (mixed blocks of 4 or 8) was used to allocate the patients. The allocation sequence was computer-generated by an external expert who did not involve in the trial. The randomization procedure was conducted through interactive web-based response system. The randomized subjects were treated with the investigational drug (fimasartan or losartan) corresponding to each treatment group for 24 weeks. After 24 weeks, all participants were administered open-labeled fimasartan and maintained in group A and group B in a single blinded manner. Antihypertensive regimen was adjusted if the patient BP does not meet the respective target SBP at each visit according to the study protocol as described elsewhere. The dose titrations were made at the discretion of the investigators [11]. The participants made visits every 12 weeks, or until the secondary endpoint occurred.

### Measurements

BP measurement was performed by a trained nurse at each center according to the standardized protocol [12]. BP was measured after 5 min of rest in a seated position at the clinic office using a validated, oscillometric sphygmomanometer (HEM-7080IC; Omron, Kyoto, Japan). The mean of three BP readings on the selected arm was used as the BP value for the visit. Blood samples were obtained after 8 h of overnight fasting. Serum creatinine was measured using an isotope-dilution mass spectrometry-tractable method, and the glomerular filtration rate was estimated using the Modification of Diet in Renal Disease (MDRD) equation [13]. The spot UACR from first-voided urine sample in the morning was used for urinary albumin excretion measurement.

### Outcomes

The primary outcomes of interest were the prespecified secondary outcomes of the FANTASTIC study, defined as the composite of cardiovascular events and renal events. Cardiovascular events were defined as non-fatal myocardial infarction, non-fatal stroke, hospitalization for heart failure, unstable angina, revascularization (coronary or peripheral), or death from cardiovascular cause. Renal events were defined as a composite of a ≥ 50% decline in eGFR from the baseline measurement or initiation of kidney replacement therapy. The secondary outcomes of interest were the individual components of the primary composite outcome (cardiovascular and renal outcomes). The events were adjudicated by an independent event-adjudication committee.

### Adverse event

An adverse event was defined as a composite of orthostatic hypotension, bradycardia, syncope, acute kidney injury (AKI), or electrolyte imbalance. Adverse laboratory measures were detected on regular or unscheduled visits. AKI was defined as a ≥ 0.3 mg/dL or ≥ 1.5-fold increase in serum creatinine level from the previous visit and a decrease in serum creatinine level at the next visit. If the alteration in renal function also fulfilled the criterion for renal outcome, the event was considered a renal outcome, not AKI. Electrolyte imbalance contains hyponatremia (Na < 130 mmol/L), hypernatremia (Na > 150 mmol/L), hypokalemia (K < 3.0 mmol/L), and hyperkalemia (K > 5.5 mmol/L).

### Sample size calculation

The sample size in this study was calculated to verify the non-inferiority of fimasartan compared with losartan in terms of reducing proteinuria [11].

### Statistical analysis

To evaluate the association between BP and clinical outcomes in patients with DKD, we designed two analytical sets. First, we compared the time to the first occurrence of the cardiorenal outcome events between the standard and strict BP control groups. Second, we compared the risk of cardiorenal events according to achieved average SBP groups (< 130, 130–140, and ≥ 140 mmHg). Achieved average BP was determined by averaging BP readings at any given visit until the event occurred. Cox proportional hazards regression model was used in the analyses. Incremental adjustments were performed in the second analytical set using variables which were selected based on the univariate analysis results with *P* < 0.10. Model 1 was a crude model. In model 2, we adjusted for age, sex, body mass index, and smoking history. We added baseline eGFR, UACR, and achieved average HbA1c in model 3. The results from multivariate hazard models are presented as hazard ratios (HRs) and 95% confidence intervals (CIs). We additionally explored the continuous and nonlinear relationship between achieved average SBP and the primary outcome using adjusted restricted cubic spline models with 5 knots placed at the 5th, 25th, 50th, 75th, and 95th percentiles. We examined the effect modification of the association of achieved average SBP with the composite cardiorenal outcome in prespecified subgroups by age (< 60 and ≥ 60 years), sex, eGFR (≥ 45 and < 45 mL/min/1.73 m^2^), UACR (< 1500 and ≥ 1500 mg/gCr), and UACR dip (≥ 30 and < 30%). UACR dip was defined as the proportion of decrease in UACR at 24 weeks compared to that at baseline. To assess the risk of adverse events according to the achieved average SBP groups, we compared the occurrence of adverse events using Cox proportional hazards model including all covariates mentioned above. Lastly, we further evaluated the association between achieved average DBP and the outcome of interest.

All analyses were performed in Stata 15.1 (Stata Corporation, College Station, TX, USA).

## Results

### Baseline characteristics and the risk of outcomes according to treatment groups

**Fig. 2:**
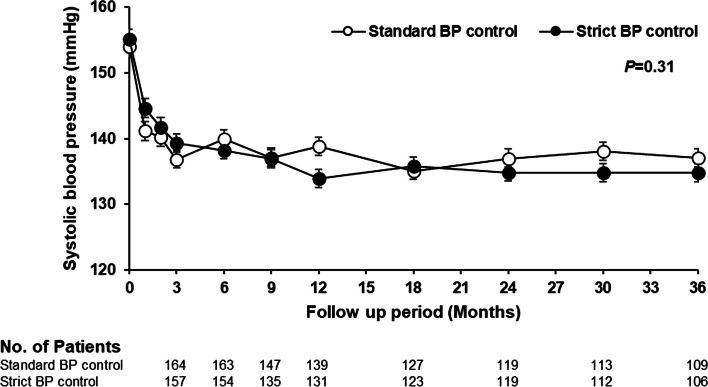
Systolic blood pressure in the two treatment groups over the course of the trial. The SBP target in the standard BP control group was less than 140 mmHg and the target in the strict BP control group was less than 130 mmHg. Abbreviations: BP, blood pressure; SBP, systolic blood pressure

Of 341 participants, the median age was 62 (interquartile range [IQR] 55–69) years; 74.2% of the participants were men. The median eGFR was 56.0 (IQR 42.0–75.0) mL/min/1.73 m^2^ and the median UACR was 929.4 (IQR 416.2–1837.6) mg/gCr. Baseline characteristics were generally similar between two treatment groups (Table 1). The two BP control strategies did not show between-group difference in SBP (*P* = 0.31) (Fig. 2). During 8657 person-months of follow-up (over a median of 2.8 years), the primary outcome event occurred in 25 participants;11 (0.25% per month) in the standard BP control group and 14 (0.33% per month) in the strict BP control group. The Kaplan–Meier curves showed similar incidence of outcomes across BP control strategies (Supplementary Fig. 1). The strict BP control group did not exhibit an improvement in terms of the primary outcome (HR 1.32; 95% CI 0.60–2.92) (Table 1).

**Table 1:** Baseline characteristics and the risk of primary outcome according to the treatment groups

	Total	BP control strategy		*P*-value
		Standard BP control	Strict BP control	
	*N* = 341	*N* = 172	*N* = 169	
**Baseline characteristics**				
Age, yr	62 (55–69)	62 (55–69)	64 (57–70)	0.12
Men, n(%)	253 (74.2)	121 (70.3)	132 (78.1)	0.10
BMI, kg/m^2^	26.8 (3.9)	27.0 (4.0)	26.6 (3.7)	0.37
Smoking status				0.62
Non-smoker	145 (42.5)	77 (44.8)	68 (40.2)	
Ex-smoker	115 (33.7)	54 (31.4)	61 (36.1)	
Current smoker	81 (23.8)	41 (23.8)	40 (23.7)	
Alcohol, n(%)	147 (43.1)	74 (43.0)	73 (43.2)	0.97
Baseline SBP, mmHg	154.6 (10.4)	154.0 (10.5)	155.2 (10.3)	0.30
Baseline DBP, mmHg	83.9 (10.0)	85.0 (9.9)	82.8 (10.1)	0.05
Achieved average SBP, mmHg	140.2 (11.7)	140.2 (11.6)	140.2 (11.9)	0.9
Achieved average DBP, mmHg	77.1 (8.6)	78.2 (8.7)	76.1 (8.4)	0.02
Serum creatinine, mg/dL	1.3 (0.4)	1.2 (0.4)	1.3 (0.4)	0.07
eGFR, mL/min per 1.73 m^2^	56.0 (42.0–75.0)	57.0 (42.0–80.5)	54.0 (42.0–72.0)	0.16
UACR, mg/gCr	929.4 (416.2–1837.6)	861.0 (393.6–1795.9)	994.6 (438.0–1979.3)	0.64
Na, mmol/L	140 (3)	140 (3)	140 (3)	0.32
K, mmol/L	4.5 (0.4)	4.5 (0.4)	4.6 (0.4)	0.19
Hemoglobin, g/dL	13.4 (1.9)	13.5 (2.0)	13.4 (1.9)	0.44
Glucose, mg/dL	157.8 (64.9)	152.4 (52.7)	163.3 (75.1)	0.12
HbA1c, %	7.0 (6.4–7.8)	7.0 (6.4–7.9)	7.0 (6.3–7.7)	0.20
Albumin, g/dL	4.1 (0.4)	4.1 (0.4)	4.1 (0.4)	0.46
Total cholesterol, mg/dL	157.5 (38.2)	157.7 (35.2)	157.3 (41.2)	0.9
LDL cholesterol, mg/dL	87.9 (34.1)	88.2 (28.9)	87.5 (38.8)	0.84
HDL cholesterol, mg/dL	46.3 (13.6)	46.6 (13.1)	45.9 (14.1)	0.62
Triglyceride, mg/dL	194.0 (148.0)	189.0 (129.1)	199.0 (165.2)	0.54
Treatment naïve patients, n(%)	16 (4.7)	8 (4.7)	8 (4.7)	0.97
No. of antihypertensive drugs (baseline)	2.9 (1.1)	2.9 (1.2)	3.0 (1.1)	0.26
ACEI/ARB, n(%)	242 (71.0)	126 (73.3)	116 (68.6)	0.35
β-blockers, n(%)	162 (47.5)	74 (43.0)	88 (52.1)	0.09
Calcium channel blockers, n(%)	297 (87.1)	147 (85.5)	150 (88.8)	0.36
Diuretics, n(%)	155 (45.5)	73 (42.4)	82 (48.5)	0.26
Peripheral vasodilator, n(%)	50 (14.7)	29 (16.9)	21 (12.4)	0.25
Statins, n(%)	326 (95.6)	164 (95.3)	162 (95.9)	0.82
Up-titration of ARB, n(%)	274 (80.4)	132 (76.7)	142 (84.0)	0.09
No. of antihypertensive drugs (end of study)	3.3 (2.1)	3.2 (2.1)	3.5 (2.1)	0.24
**Outcomes**				
Cardiovascular & Renal outcomes				
Events, n(%)	25 (7.3)	11 (6.4)	14 (8.3)	0.49
% per month	0.29	0.25	0.33	
Cardiovascular outcome				
Events	6	2	4	0.39
% per month	0.07	0.05	0.09	
Renal outcomes				
Events	19	9	10	0.75
% per month	0.22	0.20	0.24	
**Hazard ratios (95% confidence interval)**				
Cardiovascular & Renal outcomes		1.00	1.32 (0.60–2.92)	0.49
Cardiovascular outcome		1.00	2.08 (0.38–11.37)	0.40
Renal outcomes		1.00	1.15 (0.47–2.84)	0.75
**Adverse events**				
Events, n(%)	141 (41.3)	68 (39.5)	73 (43.2)	0.30

Data are expressed as mean (SD), median [interquartile range], or count (%)

*Abbreviations*: *ACEI* Angiotensin converting enzyme inhibitor, *ARB* Angiotensin receptor blocker, *BMI* Body mass index, *DBP* Diastolic blood pressure, *eGFR* Estimated glomerular filtration rate, *HDL* High-density lipoprotein, *LDL* Low-density lipoprotein, *SBP* Systolic blood pressure, *UACR* Urine albumin-to-creatinine ratio

### Baseline characteristics and the risk of outcomes according to the achieved average SBP categories

Table 2 presents the baseline characteristics according to the achieved average SBP categories. Overall, participants with higher achieved average SBP were more likely to be men, have higher baseline SBP and lower kidney function, show higher urinary albumin excretion rate, and use more antihypertensive drugs.

**Table 2:** Baseline characteristics according to the achieved average SBP categories

	Total	Achieved average SBP category		
		< 130 mmHg	130–139 mmHg	≥ 140 mmHg
	*N* = 341	*N* = 57	*N* = 132	*N* = 152
Age, yr	62 (55–69)	63 (55–68)	62 (53–68)	63 (57–70)
Men, n(%)	253 (74.2)	34 (59.6)	98 (74.2)	121 (79.6)
BMI, kg/m^2^	26.8 (3.9)	26.6 (3.7)	27.4 (4.5)	26.2 (3.3)
Smoking status				
Non-smoker	145 (42.5)	29 (50.9)	55 (41.7)	61 (40.1)
Ex-smoker	115 (33.7)	15 (26.3)	41 (31.1)	59 (38.8)
Current smoker	81 (23.8)	13 (22.8)	36 (27.3)	32 (21.1)
Alcohol, n(%)	147 (43.1)	22 (38.6)	66 (50.0)	59 (38.8)
Baseline SBP, mmHg	154.6 (10.4)	148.2 (7.5)	152.2 (8.9)	159.1 (10.6)
Baseline DBP, mmHg	83.9 (10.0)	84.7 (9.3)	84.7 (10.0)	83.0 (10.4)
Achieved average SBP, mmHg	140.2 (11.7)	124.6 (4.3)	135.2 (2.7)	150.4 (8.7)
Achieved average DBP, mmHg	77.1 (8.6)	73.9 (7.3)	76.7 (7.8)	78.7 (9.4)
Serum creatinine, mg/dL	1.3 (0.4)	1.1 (0.4)	1.2 (0.4)	1.3 (0.4)
eGFR, mL/min per 1.73 m^2^	56.0 (42.0–75.0)	58.0 (47.0–88.0)	59.5 (44.5–80.0)	53.0 (38.5–67.5)
UACR, mg/gCr	929.4 (416.2–1837.6)	752.3 (337.7–1420.4)	767.1 (351.0–1663.0)	1104.8 (566.8–2545.4)
Na, mmol/L	140 (3)	140 (3)	140 (2)	140 (3)
K, mmol/L	4.5 (0.4)	4.5 (0.4)	4.5 (0.4)	4.5 (0.5)
Hemoglobin, g/dL	13.4 (1.9)	13.7 (1.7)	13.8 (1.8)	13.0 (2.0)
Glucose, mg/dL	157.8 (64.9)	151.7 (55.3)	160.4 (61.5)	157.8 (71.1)
HbA1c, %	7.0 (6.4–7.8)	7.1 (6.6–7.9)	7.0 (6.3–7.8)	7.0 (6.3–7.8)
Albumin, g/dL	4.1 (0.4)	4.1 (0.4)	4.1 (0.4)	4.0 (0.5)
Total cholesterol, mg/dL	157.5 (38.2)	162.4 (39.3)	155.9 (38.9)	157.1 (37.2)
LDL cholesterol, mg/dL	87.9 (34.1)	91.0 (32.5)	85.3 (27.4)	88.9 (39.6)
HDL cholesterol, mg/dL	46.3 (13.6)	47.9 (15.1)	45.8 (13.4)	46.1 (13.3)
Triglyceride, mg/dL	194.0 (148.0)	182.2 (106.7)	214.3 (197.6)	180.7 (103.0)
Treatment naïve patients, n(%)	16 (4.7)	6 (10.7)	7 (5.3)	3 (2.0)
No. of antihypertensive drugs (baseline)	2.9 (1.1)	2.2 (0.9)	2.7 (1.1)	3.4 (1.1)
ACEI/ARB, n(%)	242 (71.0)	42 (75.0)	94 (70.7)	106 (69.7)
β-blockers, n(%)	162 (47.5)	12 (21.4)	54 (40.6)	96 (63.2)
Calcium channel blockers, n(%)	297 (87.1)	41 (73.2)	113 (85.0)	143 (94.1)
Diuretics, n(%)	155 (45.5)	15 (26.8)	49 (36.8)	91 (59.9)
Peripheral vasodilator, n(%)	50 (14.7)	2 (3.6)	11 (8.3)	37 (24.3)
Statins, n(%)	326 (95.6)	54 (96.4)	130 (97.7)	142 (93.4)
Up-titration of ARB, n(%)	274 (80.4)	19 (33.9)	112 (84.2)	143 (94.1)
No. of antihypertensive drugs (end of study)	3.3 (2.1)	2.6 (1.6)	3.2 (2.2)	3.7 (2.2)

Data are expressed as mean (SD), median [interquartile range], or count (%)

*Abbreviations*: *ACEI* Angiotensin converting enzyme inhibitor, *ARB* Angiotensin receptor blocker, *BMI* Body mass index, *DBP* Diastolic blood pressure, *eGFR* Estimated glomerular filtration rate, *HDL* High-density lipoprotein, *LDL* Low-density lipoprotein, *SBP* Systolic blood pressure, *UACR* Urine albumin-to-creatinine ratio

**Fig. 3:**
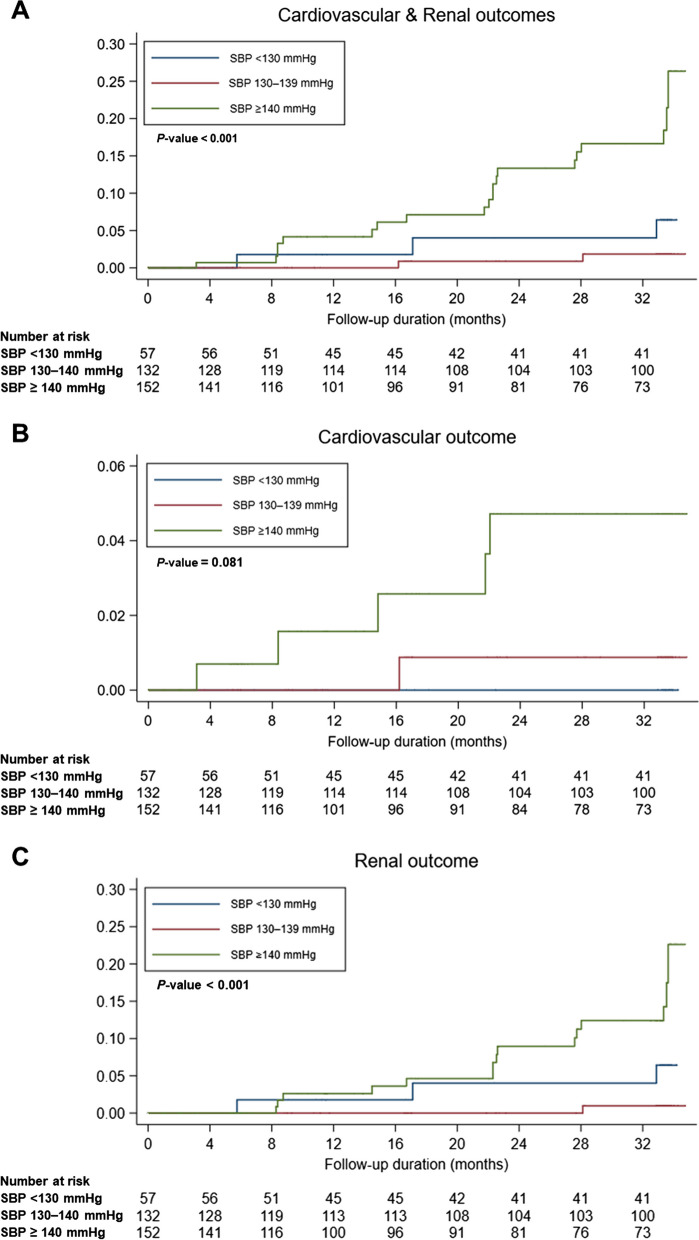
Kaplan–Meier curves for the outcomes according to the achieved average SBP categories. Kaplan–Meier curves for cardiovascular and renal outcomes (**A**), cardiovascular outcome (**B**), and renal outcome (**C**). Abbreviations: SBP, systolic blood pressure

The Kaplan–Meier curves demonstrated a significantly increased risk for cardiovascular, renal, and composite cardiorenal outcomes in individuals with an achieved average SBP > 140 mmHg compared to those of individuals with an achieved average SBP between 130–139 mmHg (Fig. 3). The event rates for the primary outcome were the highest in individuals with an achieved average SBP ≥ 140 mmHg (Supplementary Table 1).

Next, we compared the risk of primary outcomes according to achieved average SBP categories using Cox proportional hazards regression. On multivariate Cox regression analysis, an achieved average SBP of 130–139 mmHg was associated with a significant reduction in the primary outcome (HR 0.15; 95% CI 0.03–0.67) and renal outcome (HR 0.09; 95% CI 0.01–0.73) compared to those associated with an achieved average SBP ≥ 140 mmHg (model 3 in Table 3). However, ‘on treatment’ average SBP < 130 mmHg did not reduce the cardiovascular and renal outcomes compared to those associated with an SBP ≥ 140 mmHg (model 3 in Table 3) and even correlated with an increased risk for adverse kidney events compared to ‘on treatment’ average SBP of 130–139 mmHg (Supplementary Table 2). A restricted cubic spline curve for the adjusted HRs (log-transformed) for the primary and renal outcomes also showed similar results (Supplementary Fig. 2).

**Table 3:** Achieved average SBP and the risks of outcomes

Achieved average SBP category	Model 1		Model 2		Model 3	
	HR (95% CI)	*P*-value	HR (95% CI)	*P*-value	HR (95% CI)	*P*-value
**Cardiovascular & Renal outcomes**						
< 130 mmHg	0.29 (0.09–0.98)	0.05	0.28 (0.08–0.94)	0.04	0.71 (0.19–2.68)	0.61
130–139 mmHg	0.08 (0.02–0.35)	0.001	0.08 (0.02–0.36)	0.001	0.15 (0.03–0.67)	0.01
≥ 140 mmHg	1.00	-	1.00	-	1.00	-
**Cardiovascular outcome**						
< 130 mmHg	-	-	-	-	-	-
130–139 mmHg	0.18 (0.02–1.54)	0.12	0.23 (0.03–2.01)	0.19	0.29 (0.03–2.79)	0.29
≥ 140 mmHg	1.00	-	1.00	-	1.00	-
**Renal outcome**						
< 130 mmHg	0.37 (0.11–1.29)	0.12	0.32 (0.09–1.11)	0.07	1.24 (0.28–5.49)	0.78
130–139 mmHg	0.05 (0.01-.41)	0.005	0.05 (0.01–0.38)	0.004	0.09 (0.01–0.73)	0.02
≥ 140 mmHg	1.00	-	1.00	-	1.00	-

Model 1: unadjusted

Model 2: adjusted for age, sex, BMI, and smoking history

Model 3: Model 2 + eGFR, UACR, and achieved average HbA1c

*Abbreviations*: *BMI* Body mass index, *CI* Confidence interval, *eGFR* Estimated glomerular filtration rate, *HR* Hazard ratio, *SBP* Systolic blood pressure, *UACR* Urine albumin-to-creatinine ratio

### Achieved average SBP and the risk of adverse event

Overall, adverse events occurred in 141 participants. No serious adverse drug reactions or deaths were reported. The adjusted HRs (95% CIs) for adverse events were 0.92 (0.55–1.54) and 0.90 (0.61–1.31) for an achieved average SBP < 130 and 130–139 mmHg, respectively, compared to those associated with an achieved average SBP ≥ 140 mmHg (Supplementary Table 3). We repeated the analyses regarding individual components of the adverse event. The risks for the individual components were similar across achieved average SBP categories.

### Subgroup analysis

We examined whether the relationship between achieved averaged SBP and the risk of the primary outcome altered prespecified subgroups. We tested the interactions among subgroups according to age, sex, eGFR, UACR, and UACR dip (Supplementary Fig. 3). The effects of the achieved average SBP and the risk of the primary outcome were consistent across the aforementioned subgroups.

### Achieved average DBP and the risk of outcomes

Baseline characteristics according to achieved average DBP categories are described in Supplementary Table 4. Participants with higher achieved average DBP were more likely to be younger and men and have higher baseline DBP and higher kidney function. Unlike the relationship between achieved average SBP and cardiorenal outcomes, there was no difference in the risk of outcomes across ‘on treatment’ average DBP categories (Supplementary Table 5).

## Discussion

This study had several key findings. First, in treated hypertensive DKD patients, an achieved average SBP of 130–139 mmHg was associated with the lowest risk of composite cardiovascular and renal outcomes and renal outcomes. Second, for patients with an achieved SBP < 130 mmHg, there was an increase in renal outcomes compared to that observed in individuals with SBP of 130–139 mmHg. Finally, DBP was not significantly associated with cardiovascular or renal outcomes.

BP targets vary according to different guidelines for BP management [3, 14–17]. Especially, the target BP in CKD patients is a subject of controversy [18]. In the latest ACC/AHA/ASH guidelines for the management of high BP, the recommended BP target in CKD patients was below 130/80 mmHg [15]. However, the 2018 ESC/ESH guidelines for the management of arterial hypertension recommended target SBP of 130–139 mmHg and DBP of 70–79 mmHg in patients with CKD due to conflicting evidence regarding the benefit of intensive SBP lowering in the CKD patients [16]. Furthermore, the 2021 KDIGO practice guideline for BP managements suggested a target SBP < 120 mmHg in CKD patients [3]. The 2021 KDIGO guideline lowered BP target primarily based on the findings from the SPRINT study, in which intensive lowering SBP to < 120 mmHg showed improved cardiovascular outcomes and reduced death from any cause [1–3]. Although the SPRINT study revealed benefits of the lower SBP target regarding cardiovascular outcomes, the target BP recommended by the latest KDIGO guideline elicited debates, especially implementing the BP target in patients with DKD [19–21]. These disputes are partly attributed to the characteristics of the SPRINT study that excluded patients with diabetes or prior stroke and limited the power of the study to detect the effect of intensive BP lowering on kidney outcome [1, 2].

Adopting a BP target in DKD patients seems complicated because of discrepancy among results from various studies involving those patients [18]. In the Appropriate Blood Pressure Control in Diabetes (ABCD) study in hypertensive patients and normotensive ABCD study, intensive BP control strategy failed to demonstrate ameliorating creatinine clearance decline. (ABCD study in hypertensive patients, an achieved SBP of 132 mmHg *versus* 138 mmHg; normotensive ABCD study, an achieved SBP of 128 mmHg *versus* 137 mmHg) [22, 23]. It should be noted that intensive BP lowering arm, targeting SBP less than 120 mmHg, in ACCORD study did not result in a favorable cardiovascular outcome and even showed increased adverse kidney outcomes [8, 9]. In addition, a *post-hoc* analysis of the Reduction of Endpoints in Non-Insulin-Dependent Diabetes Mellitus with the Angiotensin II Antagonist Losartan (RENAAL) study revealed that an achieved SBP < 140 mmHg was associated with a decreased risk of renal endpoint, defined as a composite of doubling of serum creatinine, kidney failure with replacement therapy, or death [24]. However, an achieved SBP < 130 mmHg and 130–139 mmHg were associated with a comparable risk for renal outcomes in the study [24]. Additionally, Pohl M et al. [25] reported that a follow-up achieved SBP < 134 mmHg was associated with the lowest risk for adverse kidney outcome and an achieved SBP < 120 mmHg was associated with an increased risk of all-cause mortality in the *post-hoc* analysis of the Irbesartan Diabetic Nephropathy Trial (IDNT). These conflicting results make setting a target BP difficult in patients with DKD.

In the present study, we analyzed the effect of achieved BP on cardiovascular outcomes and renal outcomes in DKD patients. The results clearly revealed a significantly decreased risk of adverse kidney outcomes in individuals with an achieved average SBP of 130–139 mmHg compared to that in individuals with an achieved average SBP ≥ 140 mmHg. Patients with ‘on treatment’ SBP < 130 mmHg had an increased risk of kidney events compared to that in individuals with ‘on treatment’ SBP of 130–139 mmHg, suggesting a J-curve phenomenon of renal outcomes in individuals with DKD. In line with our findings, a long-term follow-up study of an ACCORD cohort showed that intensive BP lowering showed poor kidney outcomes, even though subgroup analyses of ACCORD study suggested that eGFR declines in intensive treatment arm may reflect hemodynamic changes rather than true kidney damage [9, 26]. Our results showed that even an achieved SBP < 130 mmHg, not SBP < 120 mmHg, was associated with adverse kidney events. This may be attributed to differences in characteristics of the study populations between the ACCORD study and the FANTASTIC trial. The mean eGFR and median UACR of participants in the ACCORD study were 91.6 mL/min/1.73 m^2^ and 14.3 mg/gCr, respectively, showing better kidney function and lower urinary albumin excretion rate than those in the FANTASTIC trial [8]. An interesting finding from this study was the lack of association between achieved DBP and cardiorenal outcomes. Generally, an elevated DBP is also considered an independent risk factor for adverse outcomes including kidney failure, cardiovascular disease and death, although DBP is considered less important than SBP in predicting those events [27–30]. In many studies incorporating DKD patients, DBP threshold for adverse events was usually ≥ 90 mmHg, including the RENAAL study [24, 29, 30]. However, an achieved DBP was not associated with adverse kidney events in a secondary analysis of the IDNT, similar to our findings [25]. As the age of the study population was relatively high, the baseline DBP was relatively low as shown in Table 2. Therefore, further reduction in DBP may not have had a significant effect on cardiorenal outcomes in this study.

Our study had several limitations. First, the FANTASTIC trial did not show significant between-group differences in SBP according to BP control strategy (standard BP control *versus* strict BP control). Thus, we could not analyze the effect of intensive BP lowering in DKD. This may have been due to the lack of forced titration in the study protocol for the intensive BP treatment arm, evident by the lack of difference in the number of anti-hypertensive medications in both treatment arms. As the study population consisted mostly of DKD patients with overt proteinuria, the BP was more difficult to control evidenced by the fact that the mean (SD) number of antihypertensive drugs was 2.9 (1.1) in the FANTASTIC trial, whereas it was 1.8 (1.0) in the SPRINT study. The high number of anti-hypertensive medications used in these patients, including the relatively frequent use of beta blockers, might have contributed to failure in titration of antihypertensive medications. The reluctance to use diuretics by the investigators may have contributed as well, with only half of the study population being prescribed with diuretics [31]. For this reason, we performed pooled analyses using achieved average SBP in addition to intention-to-treat analysis and showed the effect of achieved average SBP on clinical outcomes. Second, residual confounding factors may exist because the pooled analyses were retrospective in nature. This was evident as participants with an achieved SBP > 140 mmHg had lower eGFR and larger degree of albuminuria. To overcome this concern, we adjusted for covariates that might affect the outcomes. However, we cannot rule out the possibility that lower BP was achieved because the participants experienced lesser difficulty to treat hypertension, evidenced by higher eGFR, smaller degree of albuminuria and the same number of anti-hypertensive medications. However, the achieved BP in our study cannot be equivalent to the treatment target in randomized controlled trials. Therefore, our findings should be interpreted with caution. Lastly, the FATASTIC trial enrolled only Koreans with relatively advanced age, limiting the generalizability of the study. Thus, our findings cannot be directly extrapolated to DKD patients with other ethnic backgrounds or younger ages.

## Conclusion

An achieved average SBP of 130–139 mmHg was associated with a significant risk reduction in the composite of cardiovascular and kidney outcomes compared to that associated with an achieved average SBP ≥ 140 mmHg in patients with DKD. However, ‘on treatment’ SBP < 130 mmHg did not decrease the risk of composite outcomes compared to that associated with ‘on treatment’ SBP ≥ 140 mmHg and even increased the risk of adverse kidney outcomes compared to that associated with ‘on treatment’ SBP of 130–139 mmHg. Therefore, an SBP target of 130–139 mmHg may be appropriate in DKD patients for improving cardiovascular and renal outcomes.

### Supplementary Information

Supplementary Material 1.

## Data Availability

All data generated or analysed during this study are included in this published article [and its supplementary information files].

## Abbreviations

ABCD: Appropriate Blood Pressure Control in Diabetes
ACCORD: Action to Control Cardiovascular Risk in Diabetes
AKI: Acute kidney injury
ARB: Angiotensin receptor blocker
BP: Blood pressure
CI: Confidence interval
CKD: Chronic kidney disease
DBP: Diastolic blood pressure
DKD: Diabetic kidney disease
eGFR: Estimated glomerular filtration rate
FANTASTIC: FimAsartaN proTeinuriA SusTaIned reduCtion in comparison with losartan in diabetic chronic kidney disease
HR: Hazard ratio
IDNT: Irbesartan Diabetic Nephropathy Trial
KDIGO: Kidney Disease: Improving Global Outcomes
MDRD: Modification of Diet in Renal Disease
RENAAL: Non-Insulin-Dependent Diabetes Mellitus with the Angiotensin II Antagonist Losartan
SBP: Systolic blood pressure
SPRINT: Systolic Blood Pressure Intervention Trial
UACR: Urine albumin-to-creatinine ratio
