# Supplementary material for: Blood pressure control in diabetic kidney disease: a post-hoc analysis of the FANTASTIC trial
Source: Clin Hypertens. 2024 Aug 1;30:20. doi: 10.1186/s40885-024-00280-x (PMC11293031; doi:10.1186/s40885-024-00280-x)
Supplement: Supplementary file 1 — Supplementary Material 1. [file 40885_2024_280_MOESM1_ESM.docx]

**Blood Pressure Control in Diabetic Kidney Disease: a post-hoc Analysis of the FANTASTIC Trial**

Cheol Ho Park, MD, PhD,^1,*^ Soon Jun Hong, MD, PhD,^2,*^ Sung Gyun Kim, MD,PhD,^3^ Seok Joon Shin, MD, PhD,^4^ Dong Ki Kim, MD, PhD,^5^ Jung Pyo Lee, MD, PhD,^6^ Sang Youb Han, MD, PhD,^7^ Sangho Lee, MD, PhD,^8^ Jong Chul Won, MD, PhD,^9^ Young Sun Kang, MD, PhD,^10^ Jongha Park, MD, PhD,^11^ Byoung-Geun Han, MD, PhD,^12^ Ki-Ryang Na, MD, PhD,^13^ Kyu Yeon Hur, MD, PhD,^14^ Yong-Jin Kim, MD, PhD,^15^ Sungha Park, MD, PhD,^16,†^ and Tae-Hyun Yoo, MD, PhD,^1, †^

^*^These authors contributed equally to this work as co-first authors.

^†^These authors contributed equally to this work as co-corresponding authors.

^1^Department of Internal Medicine, Institute of Kidney Disease Research, Yonsei University College of Medicine, Seoul, Republic of Korea

^2^Department of Cardiology, Korea University Anam Hospital, Seoul, Republic of Korea

^3^Division of Nephrology, Hallym University Sacred Heart Hospital, Anyang, Republic of Korea

^4^Division of Nephrology, Incheon St. Mary’s Hospital, The Catholic University of Korea, Incheon, Republic of Korea

^5^Department of Internal Medicine, Seoul National University Hospital, Kidney Research

Institute, Seoul, Republic of Korea

^6^Department of Internal Medicine, Seoul National University Boramae Medical Center, Seoul, Republic of Korea

^7^Division of Nephrology, Inje University Ilsan-Paik Hospital, Goyang, Republic of Korea

^8^Department of Nephrology, Kyung Hee University Hospital at Gangdong, Seoul, Republic of Korea

^9^Division of Endocrinology and Metabolism, Inje University Sanggye Paik Hospital, Inje University School of Medicine, Seoul, Republic of Korea

^10^Division of Endocrinology, Korea University Ansan Hospital, Ansan, Republic of Korea

^11^Division of Nephrology, Ulsan University Hospital, Ulsan, Republic of Korea

^12^Division of Nephrology, Yonsei University Wonju College of Medicine, Wonju, Republic of Korea

^13^Division of Nephrology, Chungnam National University Hospital, Daejeon, Republic of Korea

^14^Division of Endocrinology and Metabolism, Samsung Medical Center, Sungkyunkwan University School of Medicine, Seoul, Republic of Korea

^15^Division of Cardiology, Seoul National University Hospital, Seoul, Republic of Korea

^16^Division of Cardiology, Severance Cardiovascular Hospital, Yonsei University College of Medicine, Seoul, Republic of Korea.

**Corresponding author:**

Sungha Park, MD, PhD

Division of Cardiology, Severance Cardiovascular Hospital, Cardiovascular Research Institute, Yonsei University College of Medicine, 50-1 Yonsei-ro, Seodaemun-gu, Seoul, 03722, Republic of Korea

E-mail: shpark0530@yuhs.ac

Tel: 82-2-2228-8455

Fax: 82-2-393-2041

Tae-Hyun Yoo, MD, PhD

Department of Internal Medicine, Institute of Kidney Disease Research, Yonsei University College of Medicine, 50-1 Yonsei-ro, Seodaemun-gu, Seoul, 03722, Republic of Korea.

E-mail: yoosy0316@yuhs.ac

Tel: 82-2-2228-1975

Fax: 82-2-393-6884

**SUPPLEMENTARY MATERIAL**

**Supplementary Table 1.** Outcome Event Rates according to Achieved Average SBP Categories

**Supplementary Table 2.** Association between Achieved Average SBP and Outcomes (Reference: Achieved average SBP 130–139 mmHg)

**Supplementary Table 3.** Adverse Events and Monitored Clinical Events

**Supplementary Table 4.** Baseline Characteristics according to Achieved Average DBP Categories

**Supplementary Table 5.** Achieved Average DBP and the Risks of Outcomes

**Supplementary Figure 1.** Kaplan-Meier Curves for the Outcomes according to the Treatment Groups

**Supplementary Figure 2.** Restricted Cubic Spline Curves for the Achieved Average SBP and the Risks of Outcomes

**Supplementary Figure 3.** Subgroup Analysis for the Achieved Average SBP and the Risk of the Primary Outcome

**Supplementary Table 1. Outcome Event Rates according to Achieved Average SBP Categories**

|  | Total | Achieved average SBP category | | | *P*-value^a^ |
| --- | --- | --- | --- | --- | --- |
|  |  | <130 mmHg | 130–139 mmHg | ≥140 mmHg |  |
| Number of subjects | 341 | 57 | 132 | 152 |  |
| Cardiovascular & Renal outcomes |  |  |  |  |  |
| Person-month | 8657.4 | 1545.3 | 3755.7 | 3356.4 |  |
| Events | 25 | 3 | 2 | 20 |  |
| Events per 1000 person-month | 2.9 | 1.9 | 0.5 | 6.0 | <0.001 |
| Cardiovascular outcome |  |  |  |  |  |
| Person-month | 8683.5 | 1548.3 | 3756.8 | 3378.5 |  |
| Events | 6 | 0 | 1 | 5 |  |
| Events per 1000 person-month | 0.7 | 0.0 | 0.3 | 1.5 | 0.08 |
| Renal outcomes |  |  |  |  |  |
| Person-month | 8651.3 | 1545.3 | 3751.3 | 3354.7 |  |
| Events | 19 | 3 | 1 | 15 |  |
| Events per 1000 person-month | 2.2 | 1.9 | 0.3 | 4.5 | <0.001 |

Abbreviations: SBP, systolic blood pressure.

^a^*P*-value based on log-rank test.

**Supplementary Table 2. Association between Achieved Average SBP and Outcomes (Reference: Achieved average SBP 130–139 mmHg)**

| Achieved average SBP category | Model 1 | | Model 2 | | Model 3 | |
| --- | --- | --- | --- | --- | --- | --- |
|  | HR (95% CI) | *P*-value | HR (95% CI) | *P*-value | HR (95% CI) | *P*-value |
| Cardiovascular & Renal outcomes | | | | | | |
| <130 mmHg | 3.51 (0.59-21.02) | 0.17 | 3.32 (0.55-19.97) | 0.19 | 4.75 (0.77-29.33) | 0.09 |
| 130–139 mmHg | 1.00 | - | 1.00 | - | 1.00 | - |
| ≥140 mmHg | 12.13 (2.83-51.93) | 0.001 | 12.05 (2.80-51.96) | 0.001 | 6.70 (1.49-30.19) | 0.01 |
|  |  |  |  |  |  |  |
| Cardiovascular outcome | | | | | | |
| <130 mmHg | - | - | - | - | - | - |
| 130–139 mmHg | 1.00 | - | 1.00 | - | 1.00 | - |
| ≥140 mmHg | 5.55 (0.65-47.59) | 0.12 | 4.30 (0.65-47.59) | 0.12 | 3.43 (0.36-32.73) | 0.29 |
|  |  |  |  |  |  |  |
| Renal outcome | | | | | | |
| <130 mmHg | 6.91 (0.72-66.52) | 0.09 | 6.32 (0.65-61.06) | 0.11 | 13.81 (1.26-151.07) | 0.03 |
| 130–139 mmHg | 1.00 | - | 1.00 | - | 1.00 | - |
| ≥140 mmHg | 18.59 (2.45-140.81) | 0.005 | 20.00 (2.63-152.31) | 0.004 | 11.17 (1.37-90.93) | 0.02 |

Model 1: unadjusted.

Model 2: adjusted for age, sex, BMI, and smoking history.

Model 3: Model 2 + eGFR, UACR, and achieved average HbA1c.

Abbreviations: BMI, body mass index; CI, confidence interval; eGFR, estimated glomerular filtration rate; HR, hazard ratio; SBP, systolic blood pressure; UACR, urine albumin-to-creatinine ratio.

**Supplementary Table 3. Adverse Events and Monitored Clinical Events**

| Achieved average SBP category | No. of patients | Event No. (%) | HR (95% CI) | *P*-value |
| --- | --- | --- | --- | --- |
| Adverse events | | | | |
| <130 mmHg | 57 | 21(36.8) | 0.92 (0.55-1.54) | 0.76 |
| 130–139 mmHg | 132 | 52 (39.4) | 0.90 (0.61-1.31) | 0.58 |
| ≥140 mmHg | 152 | 68 (44.7) | 1.00 | - |
|  |  |  |  |  |
| Orthostatic hypotension | | | | |
| <130 mmHg | 57 | 2 (3.5) | 7.37 (0.70-78.06) | 0.10 |
| 130–139 mmHg | 132 | 1 (0.8) | 0.63 (0.05-7.83) | 0.72 |
| ≥140 mmHg | 152 | 2 (1.3) | 1.00 | - |
|  |  |  |  |  |
| Bradycardia | | | | |
| <130 mmHg | 57 | 0 (0) | - | - |
| 130–139 mmHg | 132 | 0 (0) | - | - |
| ≥140 mmHg | 152 | 1 (0.7) | 1.00 | - |
|  |  |  |  |  |
| Syncope | | | | |
| <130 mmHg | 57 | 0 (0) | - |  |
| 130–139 mmHg | 132 | 1 (0.8) | 0.86 (0.04-16.96) | 0.92 |
| ≥140 mmHg | 152 | 1 (0.7) | 1.00 | - |
|  |  |  |  |  |
| Acute kidney injury | | | | |
| <130 mmHg | 57 | 9 (15.8) | 0.74 (0.35-1.59) | 0.45 |
| 130–139 mmHg | 132 | 31 (23.5) | 1.07 (0.64-1.77) | 0.80 |
| ≥140 mmHg | 152 | 36 (23.7) | 1.00 | - |
|  |  |  |  |  |
| Electrolyte imbalance^*^ | | | | |
| <130 mmHg | 57 | 16 (28.1) | 0.92 (0.51-1.68) | 0.80 |
| 130–139 mmHg | 132 | 34 (25.8) | 0.81 (0.51-1.29) | 0.38 |
| ≥140 mmHg | 152 | 49 (32.2) | 1.00 | - |
|  |  |  |  |  |
| Na < 130 mmol/L | | | | |
| <130 mmHg | 57 | 0 (0) | - | - |
| 130–139 mmHg | 132 | 2 (1.5) | 0.84 (0.05-14.73) | 0.91 |
| ≥140 mmHg | 152 | 1 (0.7) | 1.00 | - |
|  |  |  |  |  |
| Na > 150 mmol/L | | | | |
| <130 mmHg | 57 | 0 (0) | - | - |
| 130–139 mmHg | 132 | 3 (2.3) | 1.47 (0.21-10.21) | 0.70 |
| ≥140 mmHg | 152 | 2 (1.3) | 1.00 | - |
|  |  |  |  |  |
| K < 3.0 mmol/L | | | | |
| <130 mmHg | 57 | 0 (0) | - | - |
| 130–139 mmHg | 132 | 0 (0) | - | - |
| ≥140 mmHg | 152 | 1 (0.7) | 1.00 | - |
|  |  |  |  |  |
| K > 5.5 mmol/L | | | | |
| <130 mmHg | 57 | 16 (28.1) | 0.98 (0.54-1.79) | 0.96 |
| 130–139 mmHg | 132 | 32 (24.2) | 0.81 (0.51-1.29) | 0.38 |
| ≥140 mmHg | 152 | 48 (31.6) | 1.00 | - |

^*^Electrolyte imbalance contains hyponatremia (Na < 130 mmol/L), hypernatremia (Na > 150 mmol/L), hypokalemia (K < 3.0 mmol/L), and hyperkalemia (K > 5.5 mmol/L).

Abbreviations: CI, confidence interval; HR, hazard ratio; SBP, systolic blood pressure.

**Supplementary Table 4. Baseline Characteristics according to Achieved Average DBP Categories**

|  | Total | Achieved average DBP category | | |
| --- | --- | --- | --- | --- |
|  |  | <70 mmHg | 70–79 mmHg | ≥80 mmHg |
|  | N=341 | N=68 | N=144 | N=129 |
| Age, yr | 62 (55-69) | 69 (65-76) | 64 (59-70) | 55 (49-62) |
| Men, n(%) | 253 (74.2) | 45 (66.2) | 102 (70.8) | 106 (82.2) |
| BMI, kg/m^2^ | 26.8 (3.9) | 25.9 (3.9) | 26.5 (3.9) | 27.5 (3.8) |
| Smoking status |  |  |  |  |
| Non-smoker | 145 (42.5) | 31 (45.6) | 65 (45.1) | 49 (38.0) |
| Ex-smoker | 115 (33.7) | 23 (33.8) | 50 (34.7) | 42 (32.6) |
| Current smoker | 81 (23.8) | 14 (20.6) | 29 (20.1) | 38 (29.5) |
| Alcohol, n(%) | 147 (43.1) | 21 (30.9) | 62 (43.1) | 64 (49.6) |
| Baseline SBP, mmHg | 154.6 (10.4) | 155.3 (8.8) | 153.6 (10.8) | 155.4 (10.7) |
| Baseline DBP, mmHg | 83.9 (10.0) | 72.7 (6.2) | 82.0 (7.2) | 91.9 (7.4) |
| Achieved average SBP, mmHg | 140.2 (11.7) | 138.1 (11.2) | 138.7 (11.0) | 143.1 (12.2) |
| Achieved average DBP, mmHg | 77.1 (8.6) | 65.2 (4.1) | 75.1 (3.1) | 85.7 (4.7) |
| Serum creatinine, mg/dL | 1.3 (0.4) | 1.3 (0.4) | 1.3 (0.4) | 1.2 (0.4) |
| eGFR, mL/min per 1.73 m^2^ | 56.0 (42.0-75.0) | 47.0 (38.0-70.5) | 53.5 (41.5-72.5) | 61.0 (47.0-83.0) |
| UACR, mg/gCr | 929.4 (416.2-1837.6) | 819.3 (412.7-1607.5) | 924.0 (397.9-1732.9) | 995.6 (441.6-2175.5) |
| Na, mmol/L | 140 (3) | 140 (3) | 140 (2) | 140 (3) |
| K, mmol/L | 4.5 (0.4) | 4.6 (0.5) | 4.6 (0.4) | 4.5 (0.4) |
| Hemoglobin, g/dL | 13.4 (1.9) | 12.4 (1.7) | 13.3 (1.7) | 14.2 (2.0) |
| Glucose, mg/dL | 157.8 (64.9) | 163.6 (82.7) | 156.1 (61.4) | 156.7 (58.2) |
| HbA1c, % | 7.0 (6.4-7.8) | 7.1 (6.4-7.7) | 7.0 (6.4-7.8) | 7.0 (6.3-7.8) |
| Albumin, g/dL | 4.1 (0.4) | 4.0 (0.4) | 4.1 (0.4) | 4.1 (0.5) |
| Total cholesterol, mg/dL | 157.5 (38.2) | 152.3 (38.3) | 155.9 (39.8) | 162.0 (36.2) |
| LDL cholesterol, mg/dL | 87.9 (34.1) | 89.9 (47.4) | 84.7 (30.3) | 90.3 (29.5) |
| HDL cholesterol, mg/dL | 46.3 (13.6) | 44.1 (12.7) | 46.4 (15.1) | 47.2 (12.2) |
| Triglyceride, mg/dL | 194.0 (148.0) | 170.2 (103.5) | 188.0 (146.3) | 213.2 (167.1) |
| Treatment naïve patients, n(%) | 16 (4.7) | 3 (4.4) | 7 (4.9) | 6 (4.7) |
| No. of antihypertensive drugs (baseline) | 2.9 (1.1) | 2.9 (1.1) | 2.9 (1.1) | 3.0 (1.2) |
| ACEI/ARB, n(%) | 242 (71.0) | 44 (64.7) | 103 (71.5) | 95 (73.6) |
| β-blockers, n(%) | 162 (47.5) | 25 (36.8) | 70 (48.6) | 67 (51.9) |
| Calcium channel blockers, n(%) | 297 (87.1) | 59 (86.8) | 123 (85.4) | 115 (89.1) |
| Diuretics, n(%) | 155 (45.5) | 35 (51.5) | 65 (45.1) | 55 (42.6) |
| Peripheral vasodilator, n(%) | 50 (14.7) | 9 (13.2) | 16 (11.1) | 25 (19.4) |
| Statins, n(%) | 326 (95.6) | 66 (97.1) | 137 (95.1) | 123 (95.3) |
| Up-titration of ARB, n(%) | 274 (80.4) | 50 (73.5) | 111 (77.1) | 113 (87.6) |
| No. of antihypertensive drugs (end of study) | 3.3 (2.1) | 3.9 (3.0) | 3.0 (1.8) | 3.4 (2.0) |

Data are expressed as mean (SD), median [interquartile range], or count (%).

Abbreviations: ACEI, angiotensin converting enzyme inhibitor; ARB, angiotensin receptor blocker; BMI, body mass index; DBP, diastolic blood pressure; eGFR, estimated glomerular filtration rate; HDL, high-density lipoprotein; LDL, low-density lipoprotein; SBP, systolic blood pressure; UACR, urine albumin-to-creatinine ratio.

**Supplementary Table 5. Achieved Average DBP and the Risks of Outcomes**

| Achieved average DBP category | Model 1 | | Model 2 | | Model 3 | |
| --- | --- | --- | --- | --- | --- | --- |
|  | HR (95% CI) | *P*-value | HR (95% CI) | *P*-value | HR (95% CI) | *P*-value |
| Cardiovascular & Renal outcomes | | | | | | |
| <70 mmHg | 0.19 (0.02-1.48) | 0.11 | 0.20 (0.02-1.70) | 0.14 | 0.21 (0.02-2.05) | 0.18 |
| 70–79 mmHg | 1.08 (0.48-2.41) | 0.86 | 1.07 (0.44-2.60) | 0.88 | 1.20 (0.43-3.39) | 0.73 |
| ≥80 mmHg | 1.00 | - | 1.00 | - | 1.00 | - |
|  |  |  |  |  |  |  |
| Cardiovascular outcome | | | | | | |
| <70 mmHg | - | - | - | - | - | - |
| 70–79 mmHg | 0.87 (0.18-4.33) | 0.87 | 0.36 (0.06-2.33) | 0.28 | 0.26 (0.04-1.83) | 0.18 |
| ≥80 mmHg | 1.00 | - | 1.00 | - | 1.00 | - |
|  |  |  |  |  |  |  |
| Renal outcome | | | | | | |
| <70 mmHg | 0.26 (0.03-2.11) | 0.21 | 0.41 (0.04-3.83) | 0.44 | 0.74 (0.06-8.65) | 0.81 |
| 70–79 mmHg | 1.15 (0.45-2.92) | 0.77 | 1.41 (0.51-3.92) | 0.51 | 2.53 (0.68-9.37) | 0.17 |
| ≥80 mmHg | 1.00 | - | 1.00 | - | 1.00 | - |

Model 1: unadjusted.

Model 2: adjusted for age, sex, BMI, and smoking history.

Model 3: Model 2 + eGFR, UACR, and achieved average HbA1c.

Abbreviations: BMI, body mass index; CI, confidence interval; eGFR, estimated glomerular filtration rate; HR, hazard ratio; SBP, systolic blood pressure; UACR, urine albumin-to-creatinine ratio.

**Supplementary** **Figure 1. Kaplan-Meier Curves for the Outcomes according to the Treatment Groups**


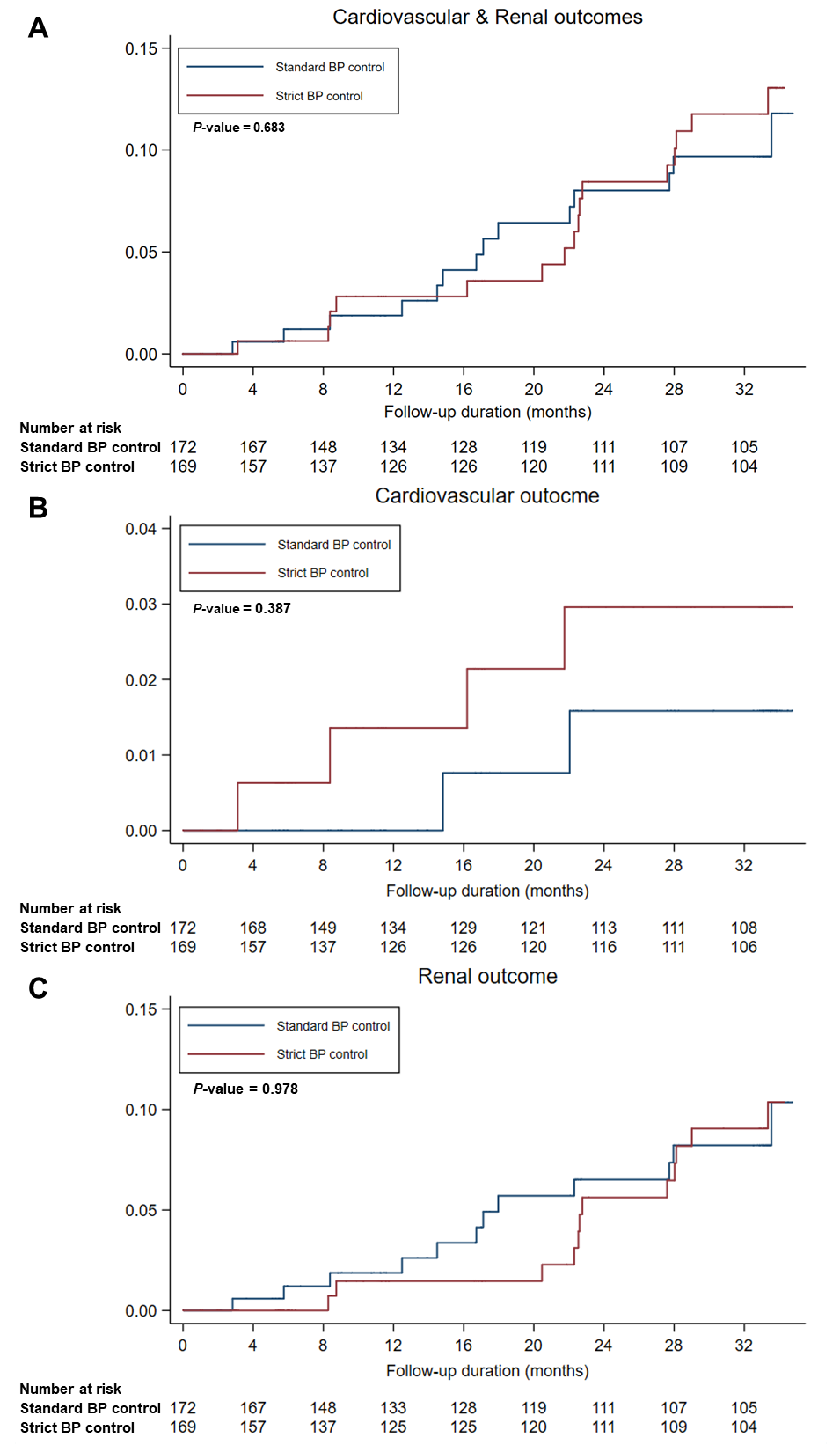


Kaplan-Meier curves for cardiovascular and renal outcomes (A), cardiovascular outcome (B), and renal outcome (C).

Abbreviations: BP; blood pressure.

**Supplementary Figure 2. Restricted Cubic Spline Curves for the Achieved Average SBP and the Risks of Outcomes**


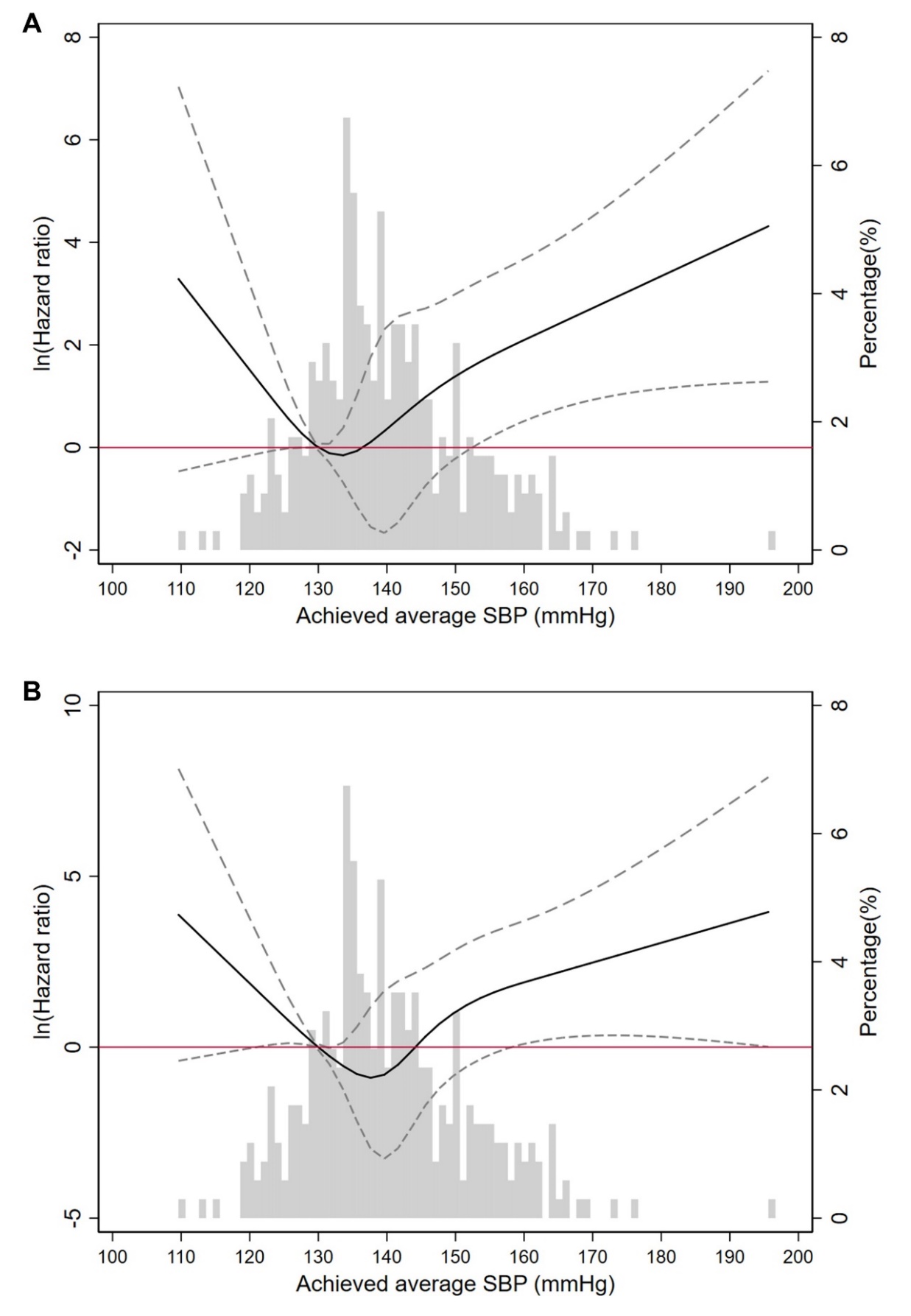


Restricted cubic spline curves for cardiovascular and renal outcomes (A) and renal outcome (B)

Abbreviation: SBP, systolic blood pressure.

**Supplementary Figure 3. Subgroup Analysis for the Achieved Average SBP and the Risk of the Primary Outcome**


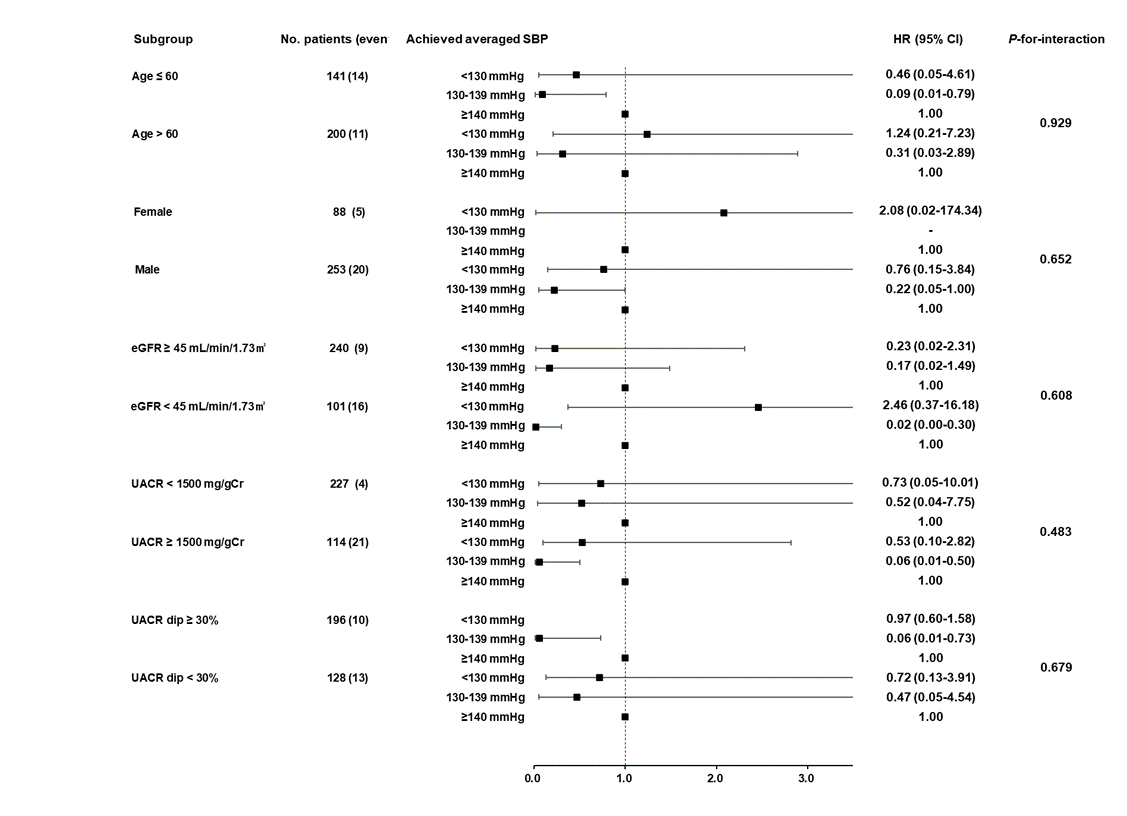


Abbreviations: CI, confidence interval; eGFR, estimated glomerular filtration rate; HR, hazard ratio; SBP, systolic blood pressure; SD, standard deviation; UACR, urinary albumin-to-creatinine ratio.
